# Health Literacy, Knowledge on Cervical Cancer and Pap Smear and Its Influence on Pre-Marital Malay Muslim Women Attitude towards Pap Smear

**DOI:** 10.31557/APJCP.2020.21.7.2021

**Published:** 2020-07

**Authors:** Nurul Nadia Baharum, Farnaza Ariffin, Mohd Rodi Isa, Su Tin Tin

**Affiliations:** 1 *Department of Primary Care Medicine, Faculty of Medicine, Universiti Teknologi MARA, Selayang Campus, 68100 Selayang, Selangor, Malaysia.*; 2 *The Maternofetal and Embryo Research Group (MatE), Faculty of Medicine, Universiti Teknologi MARA, Sungai Buloh Campus, 47000 Sungai Buloh, Selangor, Malaysia.*; 3 *Department of Population Health and Preventive Medicine, Faculty of Medicine, Universiti Teknologi MARA, Sungai Buloh Campus, 47000 Sungai Buloh, Selangor, Malaysia. *; 4 *Centre for Population Health (CePH), Department of Social and Preventive Medicine, Faculty of Medicine, University of Malaya, Kuala Lumpur, Malaysia. *; 5 *South East Asia Community Observatory (SEACO), Jeffery Cheah School of Medicine and Health Sciences, Monash University Malaysia. *

**Keywords:** Health literacy, knowledge of cervical cancer, knowledge of Pap smear, attitude towards Pap smear

## Abstract

**Background:**

Cervical cancer is preventable. In Malaysia, women are found to have good awareness of the disease and yet, the Pap smear uptake is still poor. Measuring health literacy level could explain this discrepancy. This study aims to determine the relationship between health literacy, level of knowledge of cervical cancer and Pap smear with attitude towards Pap smear among women attending pre-marital course.

**Methods:**

A cross sectional study was performed in three randomly selected centres that organised pre-marital courses. All Malay Muslim women participants aged 18 to 40 years old were recruited while non-Malaysian, illiterate, and had hysterectomy were excluded. Validated self-administered questionnaires used were European Health Literacy Questionnaire (HLS-EU-Q16 Malay) and Knowledge and attitude towards Cervical Cancer and Pap Smear Questionnaire. The mean percentage score (mean± SD) was calculated, with higher scores showed better outcomes. Multiple linear regression was used to measure the relationship of independent variables with attitude towards Pap smear.

**Results:**

A total of 417 participants were recruited with a mean age of 24.9 ± 3.56 years old. Prevalence of awareness of cervical cancer was 91.6% (n=382, 95% CI: 89.0%, 94.2%) and mean percentage score was 74.7%±7.6. Prevalence of awareness of Pap smear was 59.0% (n=246, 95% CI: 54.2%, 63.8%) and mean percentage score was 80.2% ± 6.5. The health literacy mean score was 13.3±3.6, with minimum score 0 and maximum score 16. The mean percentage score of attitudes towards Pap smear was 64.8%±9.3. Multiple linear regression analysis demonstrated significant relationship between health literacy (p=0.047) and knowledge of Pap smear (p<0.001) with attitude towards Pap smear.

**Conclusion:**

A higher health literacy with high knowledge of Pap smear improves the attitude towards Pap smear. Pre-marital course is an opportunistic platform to disseminate information to improve health literacy and knowledge of cervical cancer and Pap smear screening.

## Introduction

Cervical cancer is a preventable disease. In Malaysia, cervical cancer is the second most common form of cancer among women after breast cancer (Zaridah, 2014). Knowledge of the pathophysiology of cervical cancer and on preventive measures such as screening is well-established so that early detection can mean a potential cure (Chrysostomou et al., 2018). A review article found that the prevention of cervical cancer was the focus of much research conducted locally over the last two decades (Zaridah, 2014). Primary forms of prevention include a healthy lifestyle, smoking cessation, sexual abstinence, including safe sex practices, and human papillomavirus (HPV) vaccination. For those who are sexually active, a secondary form of prevention is through performing the pap smear to detect pre-cancerous or pre-invasive lesions. Finally, a tertiary form of prevention such as colposcopy that can detect and surgery to treat early stages of cancer. 

The pap smear is an effective and widely available cervical cancer screening tool that is performed by examining the cervical cytological components using Papanicolaou’s stain. The pap smear has been implemented as a population-based form of screening for several years in the United Kingdom (UK), the United States (US), Australia and New Zealand, leading to a drastic reduction in cervical cancer incidence and mortality (Nwabichie et al., 2017). Cervical cancer screening was introduced in Malaysia in 1969 based on an opportunistic screening model rather than a comprehensive population-based screening programme (Hausmann-Muela et al., 2003). Following this, pap smear coverage has been growing steadily from 2% in 1992 to 3.5% in 1995 and 6.2% in 1996, to eventually plateau at 47.3% in 2006, according to national data (Romli et al., 2019). 

Individual studies done in Malaysia have shown a higher documented pap smear uptake, citing 48.9% among 1,000 households in five districts of Perak (Gan and Dahlui, 2013) to 65.2% among university staff (Mohd Razi et al., 2017) and 77.6% among the indigenous people known as the Orang Asli (Indra et al., 2017). However, the overall national uptake of 47.3% is still below the required adequate uptake of 70 to 80% for a successful screening prevention programme. One of the reasons for the low coverage is that in most developing countries without a comprehensive cervical cancer screening programme, the pap smear is often accessible to only a small proportion of women who are in contact with health care services through maternal, child health or family planning clinics (Nwabichie et al., 2017). 

Following this low national uptake of the pap smear, further studies were conducted to identify other possible contributing factors, including knowledge and attitudes towards pap smear practices. Most studies concluded that good knowledge and a good attitude lead to higher uptake. Other sociodemographic factors such as older age, higher education levels, marital status, higher parity and higher income are factors that have positively influenced pap smear uptake (Dunn and Tan, 2010; Waringin Oon et al., 2010; Abdullah et al., 2011; Abdullah and Su, 2013). However, some studies found discrepancies among Malaysian women who have good knowledge of cervical cancer and yet for whom the screening uptake is still poor. This led to qualitative studies to identify barriers towards the pap smear (Wong et al., 2009; Abdullah and Su, 2010; Waringin Oon et al., 2010). Despite being the largest ethnic group in Malaysia, Malay women have a lower prevalence of screening uptake compared to Chinese women (Dunn and Tan, 2010). The main barriers identified in the Malay Muslim women’s community include embarrassment, fear of pain, a fatalistic attitude towards cancer diagnosis and an undervaluation of their health needs versus the needs of their family (Wong et al., 2009; Waringin Oon et al., 2010). Therefore, it is important to study this population further. 

Health literacy (HL) is the ability to assess and access knowledge in deciding on health matters. Having sufficient HL can improve one’s ability to acquire knowledge and understanding of a health matter (Abdullah and Su, 2013; Tung et al., 2016; Karimy et al., 2017). A systematic review published in 2016 highlighted the fact that health literacy was associated with reproductive health knowledge, behaviours, and outcomes across a spectrum of topics including cervical cancer screening (Kilfoyle et al., 2016). This study aims to identify the relationship between health literacy, knowledge of cervical cancer and pap smear, and attitudes towards the pap smear among pre-marital Malay Muslim women. 

## Materials and Methods

A cross-sectional study was conducted among Malay Muslim women attending a pre-marital course organised by the state government religious department. The data collection period was from June to September 2018 using two pre-existing translated and validated Malay version questionnaires: the European Health Literacy Questionnaire (HLS EU 16) (Chinna, 2014) and the Malay Knowledge, Attitude towards Cervical Cancer and Pap Smear Questionnaire (Idris, 2015).

*Study setting and population*

The study was conducted in three randomly selected state religious district centres from seven available centres that conducted the pre-marital course every weekend. The pre-marital course is a two-day weekend course compulsory for all Muslim couples applying for marriage via the state government religious department. It is also implemented in other countries with Muslim populations such as Iran (Yazdanpanah et al., 2014). This initiative was implemented in 1996 and the standardised module was introduced by the Department of Islamic Development Malaysia and is governed by each state according to districts (http://www.jais.gov.my/article/jadual-kursus-pra-perkahwinan-anjuran-pejabat-agama-islam, 2017). During this study, the health module did not include cervical cancer screening information. 

The study population was pre-marital Malay Muslim women. This population was chosen because there is a gap in targeting pre-marital women who normally do not have contact with health services, and Malay Muslim were chosen because studies have found that pap smear practice is low among this group, which often has barriers towards the pap smear (Wong et al., 2009; Dunn and Tan, 2010; Waringin Oon et al., 2010). Inclusion criteria included all Malay Muslim women attending the pre-marital course during the study period, aged between 18 to 35 years old, and who gave voluntary informed consent. The exclusion criteria included those who were non-Malaysian, those with a history of hysterectomy, those unable to understand the Malay language or unable to read or write, and those with a mental illness. 

*Sample size determination and sampling method*

All Malay Muslim women who attended the premarital course at the three randomly selected centres organised by the state government religious department during the data collection days were invited to participate in the study. Participation was voluntary and confidentiality was assured. The participants were approached on the first day of the pre-marital course and briefed on the purpose of the study. Those who agreed to participate were asked to sign a written consent form. The participants were then given the questionnaire, which consisted of questions about sociodemographic details, health literacy, knowledge and attitudes on cervical cancer and the pap smear. An estimated sample size of 420 participants was calculated using Open Epi and based on a 51.2% prevalence rate of adequate knowledge on the pap smear among young women in Malaysia (Al-Naggar et al., 2010). By taking α = 0.05, 80% of the power of the study and a prevalence of 51.2%, the minimum sample size was 382 and, with a 10% attrition rate, the final estimated sample size was 420. 

*Study instruments*

Two validated self-administered questionnaires used in this study were the HLS-EU-16 Malay Version and the Malay knowledge and attitude on cervical cancer and pap smear Questionnaire. 

The HLS-EU-16 Malay Version has a total of 16 items and three sub-domains as follows: Health Care (HC), Disease Prevention (DP) and Health Promotion (HP). It is shorter than the original 47-item European Health Literacy Questionnaire (HLS-EU-Q47) and has been validated in six Asian countries including Malaysia (Sørensen et al., 2013; Duong et al., 2017). The short version of HLS-EU-Q16 is also suitable to be used like its original (Chinna, 2014). Factor analysis of the Malay HLS EU Q16 sub-domains showed a good convergent validity of 0.408, 0.367 and 0.496, respectively, and discriminant validity of 0.408, 0.637 and 0.496, respectively. Its internal consistencies for reliability analysis showed 0.779 for HC, 0.775 for DP and 0.795 for HP. The HLS-EU-16 measures health literacy levels using a Likert scale from 1 to 4. The items were scored by the respondents choosing a number where the higher value indicated a better level of health literacy: (1 = Very difficult), (2 = Moderately difficult), (3 = Fairly easy) and (4 = Very easy). The scores were then categorised dichotomously whereby both difficult categories (‘Moderately difficult’ and ‘Very difficult’) were coded with 0 (zero) and the easy categories (‘Fairly easy’ and ‘Very easy’) were coded with 1 (one). The total minimum score was 0 and the maximum score was 16. Due to the empirically skewed distribution of the scale values, the differentiation between sufficient and excellent HL was not advisable. Therefore, only three levels for the scale have been defined: inadequate HL (0–8), problematic HL (9–12) and sufficient HL (13–16). In this study, the mean score was also calculated to allow uniformity with other variables.

The Knowledge and Attitude of Cervical Cancer and Pap Smear Questionnaire (Idris et al., 2015) was developed and validated in the Malay language The questionnaire was divided into three sections: knowledge of cervical cancer (Cronbach’s alpha 0.720), knowledge of the pap smear (0.722) and attitude towards pap smear screening (Cronbach’s alpha 0.703). In the section on knowledge of cervical cancer and the pap smear, the first question was on awareness of cervical cancer and the pap smear with a possible answer of either ‘Yes’ or ‘No’. If the participant answered ‘Yes’, then they were asked where they obtained the source of information, and this was followed by 14 knowledge items on cervical cancer and nine knowledge items on the pap smear. The knowledge items were scored using three categories consisting of ‘Correct’ = 3 points, ‘Don’t know’ = 2 points or ‘Wrong’ = 1 point. The total score for knowledge on cervical cancer was calculated as a ‘percentage score’ by dividing the score by the maximum score (42) and multiplying the result by 100. The total score for knowledge on the pap smear was calculated as a ‘percentage score’ by dividing the score by the maximum score (27) and multiplying the result by 100. A higher total score indicated better knowledge regarding cervical cancer and the pap smear. Attitude towards the pap smear screening component was assessed using a 5-point Likert scale ranging from Strongly disagree = 1, Disagree = 2, Neutral = 3, Agree = 4, and Strongly agree = 5. There were 13 items and negative statements were reverse coded. The total score was calculated into a ‘percentage score’ by dividing the score by the maximum score (65) and multiplying it by 100. A higher total score indicated a better attitude towards the pap smear. The mean percentage score for knowledge of cervical cancer, knowledge of the pap smear and attitude towards the pap smear was calculated to allow for uniformity in this study.

*Data analysis*

The data collected were entered and analysed using SPSS version 24. The frequency distribution, measures of central tendencies and measures of distribution were produced. For the normally distributed data, continuous variables were presented by mean and standard deviation (SD) and categorical variables were presented by absolute numbers and percentages. Total mean scores were calculated for the different sections. The relationship between independent variables, including sociodemographic factors, health literacy score, knowledge of the pap smear, knowledge of cervical cancer and the dependent variable (attitude towards the pap smear) were analysed using a simple linear regression (SLR) and all independent variables with p-value <0.05 were included in the multiple linear regression (MLR) using the stepwise method. Any significant variables in the MLR were checked for interaction and multicollinearity. The model was then tested for homoscedasticity to test for model fitness. The model was also checked for linearity by plotting the scatter plot between residuals and predicted values. 

## Results

A total of 484 participants’ responses were collected and 67 were excluded due to incomplete or missing data. The final sample of 417 participants was analysed with a mean age ± SD of 24.88 ± 3.56. 

Table 1 highlights the sociodemographic details of the participants. Most of the participants were single, completed their education up to either the diploma or degree level and unemployed with a monthly income of < RM 3,000. The majority had no sexually transmitted illness, were non-smokers and either had not had an HPV vaccination or were unsure. Further, 91.6% were aware of cervical cancer but only 56% were aware of the pap smear. Among those who were aware of cervical cancer and the pap smear, the most common sources of information were mass media, the internet and reading books or magazines. The least common sources were teachers and healthcare workers.

Table 2 shows that the participants attending the pre-marital course had a good level of knowledge of cervical cancer and the pap smear, health literacy scores and attitude towards the pap smear.

*Reported results on knowledge of cervical cancer*

Most of the respondents (87.4%) agreed that cervical cancer is common among women in Malaysia. However, 56.5% were unsure and 8.4% were unaware that cervical cancer has a pre-cancerous state that can be detected early. About half of the respondents were either unsure or unaware regarding the symptoms of cervical cancer, including dyspareunia (51.9%), intermenstrual bleeding (44.2%) and foul-smelling vaginal discharge (40.3%). On average, about half of the respondents were unaware of the risk factors of cervical cancer, which are early age of sexual activity (77.4%), AIDS (69.4%), other gynaecological cancer (66.8%), smoking (62.2%), HPV (55.5%), multiple sexual partners (53.8%) and family history of cervical cancer (49.2%). Meanwhile, 82.8% and 82.9% of respondents were unaware that fatty foods and previous miscarriages are also risks for cervical cancer, respectively. 

*Reported results on knowledge of the pap smear*

Most of the respondents (89.4%) agreed that the pap smear is for the early detection of cervical cancer but only 64% were aware that it is not to detect HIV. A majority (73.4%) knew that any previous sexual intercourse would warrant a pap smear. However, only 56.5% knew that it is warranted in those who are married but nulliparous, while only 38.1% knew that it is also required for post-menopausal women. Regarding the pap smear procedure, 65.6% of respondents were aware that it involved taking cells from the cervix. Most respondents had the misconception that they had to avoid vaginal cream (70.7%) and to abstain from sexual intercourse (67.1%) prior to the procedure. They were not aware that conventional pap smears are performed a week after the first day of menstruation (67.1%).

*Reported results on health literacy*

Almost all (90.9%) of the respondents understood the need for screening and understood warnings on unhealthy behaviour. A majority found it easy to find information on general health (72.9%) and mental health (74.3%). They felt it was easy to get professional assistance if unwell (79.1%). Most of them felt it was easy to understand doctors’ advice (83.7%), family or friends’ advice (86.3%) and prescription information (90.6%). Further, 80.6% felt that they were able to seek a second opinion and 80.1% felt that making health decisions on their own was feasible. Also, 90.4% of the participants understood health risk behaviour reduction advice and early screening as a form of cancer prevention. Most participants felt it was easy to understand health information through mass media (84.9%), to evaluate the reliability of health information broadcasted by media (78.4%) and to make good health choices from it (75.8%). They also felt it was easy to look for information on activities to aid their mental health (85.1%) and capable to evaluate their own health behaviours (83.7%). 

*Reported results on attitudes towards the pap smear*

Most of the respondents did not find performing a pap smear a burden (72.5%) but they agreed that they feared a positive result (62.6%). Further, 42.9% were concerned about the equipment used for the pap smear and 53.9% were worried about the pain or discomfort of the procedure. The respondents were more willing to get pap smears done if they were instructed by government officials (42.8%), encouraged by spouses (44.6%), present with symptoms (50.1%) or given advice by doctors (75.1%). The majority (82.3%) believed there is a need for early screening to prevent cervical cancer and did not mind spending time (71.9%) or money on the procedure (73.8%). A majority believed they would screen to prevent cervical cancer (84.8%), even if they were healthy (56.3%), and most believed that it is preventable (86.8%).

Based on Table 3, the variables with p-values of less than 0.25 were further analysed in a multiple linear regression, and were as follows: age, educational status (between primary school and masters or Ph.D.), employment status, HPV immunisation, knowledge of cervical cancer, knowledge of the pap smear and health literacy. There is a positive significant linear relationship between knowledge of the pap smear, health literacy and attitude towards the pap smear. 

**Table 1:** Sociodemographic Details of the Participants

Variable	N	Frequency, n (%)
Marital status	415	
Single		411 (99.0%)
Divorced		4 (1.0%)
Educational status	417	
Primary school		10 (2.4%)
Secondary school		108 (25.9%)
Certificate / diploma		140 (33.6%)
Degree		145 (34.8%)
Masters / PhD		14 (3.4%)
Employment status	417	
Employed		58 (13.9%)
Unemployed		359 (86.1%)
Monthly income	417	
<RM3000		362 (86.8%)
≥RM3000		55 (13.2%)
History of previous STD	417	
Yes		3 (0.7%)
No		396 (95.0%)
Unsure		18 (4.3%)
Active or passive smoker	417	
Yes		28 (6.7%)
No		389 (93.3%)
HPV immunisation	417	
Yes		109 (26.1%)
No		160 (38.1%)
Unsure		149 (35.7%)

**Table 2:** Mean Score of the Respondents for Knowledge of Cervical Cancer, Knowledge of the Pap Smear, Health Literacy and Attitude towards the Pap Smear

Variable	Total mean score ± SD	Maximum score	Minimum score
Knowledge of cervical cancer, %	74.69 ± 7.55	95.2	54.8
Knowledge of pap smear, %	80.2 ± 9.5	100	51.9
*Health literacy, mean	13.3 ± 3.6	16	0
Attitude towards pap smear, %	64.8 ± 9.26	98	48

**Table 3:** Simple Linear Regression (SLR) and Multiple Linear Regression (MLR) for Predictors of Attitude towards the Pap Smear

Variable	SLR^a^	p-value	MLR^b^	*P*-value
	b^c^(95%CI)		Adj.B^d^ (95%CI)	
Age (years)	0.42 (0.17, 0.67)	<0.001*	-	-
Marital status:				
Single	Reference		-	-
Divorced	-0.59 (-9.72, 854)	0.09		
Educational status:				
Primary school	Reference		-	-
Secondary school	-4.36 (-9.29, 0.57)	0.08		
Certificate / Diploma	-1.00 (-2.74, 0.73)	0.26		
Degree	0.30 (-1.31, 1.92)	0.72		
Master / Ph.D.	1.01 (-0.59, 2.61)	0.210*		
Employ status:				
Unemployed	Reference		-	-
Employed	1.83 (-0.80, 4.46)	0.170*		
Previous STD status:				
Yes	Reference		-	-
No	-3.76 (-12.73, 5.20)	0.41		
Unsure	-0.87 (-4.60, 2.86)	0.65		
Active or passive smoker:				
Yes	Reference		-	-
No	-1.17 (2.04, 5.60)	0.26		
HPV immunisation:				
Yes	Reference		-	-
No	0.14 (-1.59, 1.86)	0.88		
Unsure	-1.17 (-2.75, 0.41)	0.150*		
Knowledge of cervical cancer	0.05 (0.01, 0.09)	0.014*	-	-
Knowledge of pap smear	0.09 (0.01, 0.18)	<0.001*	0.06 (0.03, 0.08)	<0.001*
Health literacy	0.45 (0.20, 0.69)	<0.001*	0.31 (0.06, 0.56)	0.014*

Notes: ^a^, Simple Linear Regression; ^b^, Multiple linear regression (R2=0.081; the model reasonably fits well; model assumptions are met; thAere is no interaction between independent variable and no multicollinearity problem). Stepwise method; ^c^, Crude regression coefficient; ^d^, Adjusted regression coefficient; *, Statistically significant at 0.05 level.

## Discussion

This study targeted younger women who were about to embark on marriage to identify their health literacy, knowledge on cervical cancer and pap smears and their attitudes towards pap smears. The mean age of the women who attended the pre-marital course was 24.9 years old. It is known that the median age of women getting married in Malaysia is around 26 years old (Department of Statistics Malaysia, 2018). All the women had received a formal education, at least up to primary school, which is reflected in the 96.3% literacy rate of women in Malaysia (Department of Statistics Malaysia, 2018). The majority were unemployed and still studying. Interestingly, only 26.1% were aware whether they had had an HPV vaccination, and some were unsure. The HPV vaccination was introduced into the immunisation schedule in the year 2010 in Malaysia, targeting schoolgirls aged 13 years old (Muhammad et al, 2018). Therefore, some of these women would have had the HPV vaccination as a primary preventive measure against cervical cancer. 

The majority of the pre-marital Malay Muslim women in this study were aware of cervical cancer but only half were aware of the pap smear. This meant that despite cervical cancer awareness, many were still unaware of the preventive measures, thus leading to the low pap smear uptake. It is important for women to be aware that sexual relationships pose the main risk for cervical cancer. It is estimated that 4.8% of Malaysia’s adolescents had already experienced sexual intercourse (Mahmud, 2014). Cervical screening in Malaysia is recommended between the ages of 20 to 65 years old (Ministry of Health, 2015) and for those who have had sexual intercourse. Therefore, women should be made aware and educated on healthy sexual behaviour and cervical cancer screening as soon as they are sexually active. Furthermore, those who were aware of cervical cancer and pap smear testing stated that the internet, mass media and reading were their preferred sources of information. These sources are one-way forms of communication and do not allow for discussion, feedback, or assessment of understanding. Other studies have shown that the preferred choices of information for young people are mass media, social media, and the internet (Maharajan et al., 2015). In contrast, this study shows that interactive sources such as teachers, health care workers, friends or families were not their preferred sources of information. This is concerning because discussion and feedback are important to improving the quality of knowledge and discerning between facts and misconceptions. 

The knowledge of cervical cancer in this study was average compared to other Malaysian studies, in which knowledge varied from poor to good (Baskaran et al., 2013; Gan and Dahlui, 2013; Indra et al., 2017b). Specifically, there was a lack of knowledge about the pre-cancerous stage, which is the most important aspect of cervical cancer screening. The role of screening is to identify the pre-cancerous stage for early detection and intervention. The women in this study had adequate knowledge of the symptoms of cervical cancer, and this is known as a positive predictor for high pap smear uptake (Gan and Dahlui, 2013). However, most were unsure of the risks of getting cervical cancer, which include having multiple sexual partners, smoking, having AIDS, having an HPV infection, and having sexual intercourse at an early age. This is important because having comprehensive knowledge of the symptoms as well as the risks of cervical cancer encourages women to get themselves screened if they are at risk (Indra et al., 2017b). It was found that women in Malaysia still have minimal awareness of cervical cancer preventive methods, which include HPV vaccination, healthy diet, and cessation of smoking (Seng et al., 2018).

In this study, the women’s knowledge of pap smears was good, especially regarding the medical indications for the pap smear and the target population. This was similar to other Malaysian studies, citing good knowledge on the pap smear (Waringin Oon et al., 2010; Maharajan et al., 2015; Idris, 2015; Mohd Razi et al., 2017). However, the majority were still unaware of the actual pap smear procedures. This contrasts with a study of an Orang Asli community, where more than 60% of the participants were able to answer correctly about the pap smear procedure (Indra et al., 2017). The most glaring explanation is the difference in marital status, where the Orang Asli study participants were mostly married, as opposed to this study population, which was completely unmarried. Hence, women should be educated with detailed and accurate information on pap smear procedures prior to marriage, as studies have shown that barriers regarding the procedure can influence their attitude towards the pap smear (Dunn and Tan, 2010). 

This study found that the women showed an average attitude score towards the pap smear test despite having good knowledge. In a previous study, Malay Muslim women stated ‘embarrassment’ as a barrier (Dunn and Tan, 2010). Other barriers have included fear of losing their virginity and fear of pain during the procedure, which needs to be addressed during knowledge dissemination on cervical cancer screening (Wong et al., 2009; Waringin et al., 2010). A study done in Oman, where the majority are Muslim, also cited similar barriers towards cervical cancer screening, such as fear and embarrassment. It also reported that unmarried, widowed, divorced, or separated participants had more perceived barriers but did not conclude any cultural-linked or religious factors that contributed towards these barriers (Al-Azri et al., 2016).

This study concluded that improving knowledge of the pap smear and general health literacy would contribute to a better attitude towards the pap smear. The health literacy rate of women in this study was sufficient. Generally, those with good health literacy tended to use their health services actively and were more forthcoming during consultations with a doctor (Duong et al., 2017). Studies have shown that improvement in health literacy would improve attitudes towards the pap smear and make it more likely to have it done (Mazor et al., 2014). Specific information regarding the indications, the procedure and the results of the test would assist those who have never had a pap smear before to have a better attitude towards the procedure. In this study, there was no significant relationship between social demographic factors and attitudes towards the pap smear. This contrasts with other studies that found age, parity, socio-economic status and educational background as strongly associated factors for a better attitude and pap smear uptake (Gan and Dahlui, 2013; Zaridah, 2014).

Although pap smear screening is available in Malaysia, due to opportunistic-based screening, it relies heavily on the initiative of the individual woman or healthcare professionals to ensure the woman commits to the pap smear screening schedule. Opportunistic screening tends to capture certain members of the population, typically those who have frequent contact with healthcare professionals, such as those who attend maternal and child health or family planning clinics. This inadvertently leads to heterogenous and uneven coverage (Chrysostomou et al., 2018). This study shows the importance of targeting women in other settings such as pre-marital courses, especially since this study proves that their awareness of the pap smear is low and that improving knowledge of the pap smear and health literacy would improve attitudes towards the pap smear among these women. Other avenues for targeting young women include HPV vaccination programmes in school where girls can interact with healthcare workers and teachers to obtain accurate information on women’s and reproductive health rather than relying on the internet and social media (Mohd Jalani et al., 2016).

In conclusion, this study is a novelty as it is the first one to utilise a pre-marital course as a platform to assess knowledge on cervical cancer, knowledge on the pap smear and attitude towards the pap smear. It is also the first to utilise health literacy in this target population, using a standardised questionnaire that allows for a comparison to similar international studies. One limitation is that this study was conducted at pre-marital courses organised by the state government religious department, hence making the findings applicable to this cohort of women only and not generalisable for all women in Malaysia. Also, it may be difficult to avoid bias when assessing attitudes towards the pap smear in women who are pre-marital and who have potentially never had a pap smear done previously. 
